# Case Report: Solitary fibrous tumor of the right temporal skull base with bone destruction and postoperative recurrence

**DOI:** 10.3389/fsurg.2026.1955012

**Published:** 2026-10-20

**Authors:** Shuangyue Shen, Changli Han, Zixiao Li, Zhiyuan Cai, Zihao Jiang, Bohan Xiong, Yubo Wang, Zhichun Wang, Guangfu Di

**Affiliations:** Department of Neurosurgery, The First Affiliated Hospital of Wannan Medical College, Yijishan Hospital of Wannan Medical College, Wuhu, China

**Keywords:** bone destruction, case report, MDM2, recurrence, skull base, solitary fibrous tumor, STAT6, temporal bone

## Abstract

**Background:**

Solitary fibrous tumor (SFT) is a rare fibroblastic mesenchymal neoplasm of the central nervous system, and temporal skull-base lesions with destructive osseous change and extension toward the middle-ear region are uncommon.

**Case presentation:**

A 60-year-old woman presented with acute right peripheral facial weakness. CT demonstrated expansile mixed lytic-sclerotic destruction of the right temporal bone and middle cranial fossa floor, and MRI showed a 46 × 38 × 36 mm lobulated mass with heterogeneous signal, irregular peripheral and nodular enhancement, central nonenhancing necrotic or cystic change, and adjacent temporal-lobe edema. Right frontotemporal microsurgical resection achieved gross-total macroscopic removal, although piecemeal resection rendered microscopic margins unassessable. Histology and immunohistochemistry supported SFT, with 4 mitoses/10 high-power fields, focal necrosis, nuclear STAT6 expression, weak partial CD34 expression, MDM2 immunoreactivity, and a Ki-67 labeling index of approximately 40%. Review of the available sections showed no morphologically distinct high-grade sarcomatous component. Under the 2021 CNS WHO framework, the documented mitotic count corresponds most closely to grade 1. Postoperative radiotherapy was recommended but declined. Telephone follow-up indicated that outside-hospital MRI reportedly showed tumor recurrence at 18 months and further enlargement at 27 months. The patient also reported that outside examinations showed no metastatic disease in the lungs, liver, or skeleton. During later follow-up, the family expressed concern that the patient might not tolerate another major surgical intervention, and continued clinical and imaging follow-up was recommended.

**Conclusion:**

This case illustrates that a documented mitotic count within the CNS WHO grade 1 range does not exclude clinically significant local recurrence. In skull-base SFT, formal pathologic grading should be interpreted together with anatomic extent, margin assessability, proliferative features, and the certainty of local control when planning postoperative management and surveillance.

## 1 Introduction

Solitary fibrous tumor (SFT) is an uncommon fibroblastic mesenchymal neoplasm characterized by spindle-to-ovoid cells, a patternless architecture, collagenous stroma, and branching thin-walled vessels. Although originally described in the pleura, SFT can arise at many anatomic sites, including the central nervous system (CNS) (1). Intracranial SFTs are typically dural-based and may mimic meningioma or other hypervascular skull-base lesions on imaging. Rare temporal skull-base examples can cross anatomic compartments and create substantial operative and diagnostic challenges (2, 3).

The 2021 World Health Organization (WHO) Classification of Tumors of the Central Nervous System recognizes SFT as a single entity and grades CNS SFT according to mitotic activity and necrosis (4). The defining molecular alteration is an NAB2::STAT6 fusion, and nuclear STAT6 immunoreactivity is a highly useful surrogate marker, whereas CD34 is supportive but less specific (5–8).

We report a primary meningeal/CNS SFT centered on the right temporal skull base with intradural and extradural components, marked destructive change of the adjacent temporal bone, involvement of the tegmen tympani, and extension toward the middle-ear region. The case is clinically instructive because the tumor combined destructive skull-base anatomy and a high Ki-67 labeling index with a documented mitotic count that falls within the CNS WHO grade 1 range, yet recurrence was reported during follow-up. This clinicopathologic discordance provides an opportunity to discuss the limits of grade-based risk assessment, the meaning of gross-total macroscopic resection in a piecemeal skull-base operation, and the implications for postoperative radiotherapy and long-term surveillance.

## 2 Case description

### 2.1 Clinical presentation and imaging assessment

A 60-year-old woman was admitted with sudden-onset right facial weakness that had developed approximately 1 day before admission. She denied headache, dizziness, nausea, vomiting, seizures, or other acute neurological symptoms. Neurological examination demonstrated loss of the right forehead wrinkles, incomplete closure of the right eye, flattening of the right nasolabial fold, leftward deviation of the mouth, and air leakage from the right side during cheek puffing. Involvement of both the upper and lower facial muscles was consistent with a right peripheral facial nerve palsy**,** which was also supported by neurological consultation. Limb strength, muscle tone, and sensation were preserved, and no pathological reflexes or meningeal signs were identified. No additional cranial-nerve deficit was documented on the available admission neurological examination. A formal House–Brackmann grade was not recorded at presentation or during follow-up and therefore could not be reliably assigned.

The available records documented no previous malignancy or other major comorbidity relevant to the present tumor or its management. No previous tumor-directed surgery, radiotherapy, or systemic therapy had been performed. No medication relevant to the current presentation was documented. Her family history was unremarkable for similar tumors or known hereditary neoplastic disorders, and no relevant psychosocial factor affecting the initial diagnostic or therapeutic decision-making was identified. A formal otologic examination, otoscopy, and standardized preoperative or postoperative audiometry were not performed; therefore, objective otoscopic findings and quantitative hearing data were unavailable.

Noncontrast computed tomography (CT) demonstrated an irregular soft-tissue mass centered at the right temporal skull base with intracranial extension. Bone-window CT showed expansile mixed lytic and sclerotic destruction of the squamous temporal bone and middle cranial fossa floor, with cortical disruption, residual bony septa, and patchy mineralization. Magnetic resonance imaging (MRI) demonstrated a 46 × 38 × 36 mm lobulated mass centered on the right middle cranial fossa floor. The lesion was iso- to mildly hypointense on T1-weighted imaging and heterogeneously hyperintense on T2-weighted imaging. Diffusion-weighted imaging showed no convincing diffuse diffusion restriction. Contrast-enhanced T1-weighted imaging demonstrated irregular peripheral and nodular enhancement surrounding a central nonenhancing necrotic or cystic component. The lesion was contiguous with the destructive temporal-bone abnormality and compressed the adjacent temporal lobe, with focal surrounding edema but no substantial midline shift (Figure 1). No obvious encasement or direct involvement of a major intracranial vessel was identified on routine preoperative imaging. Initial systemic staging imaging of the lungs, liver, and skeleton showed no evidence of distant metastatic disease.

**Figure 1:**
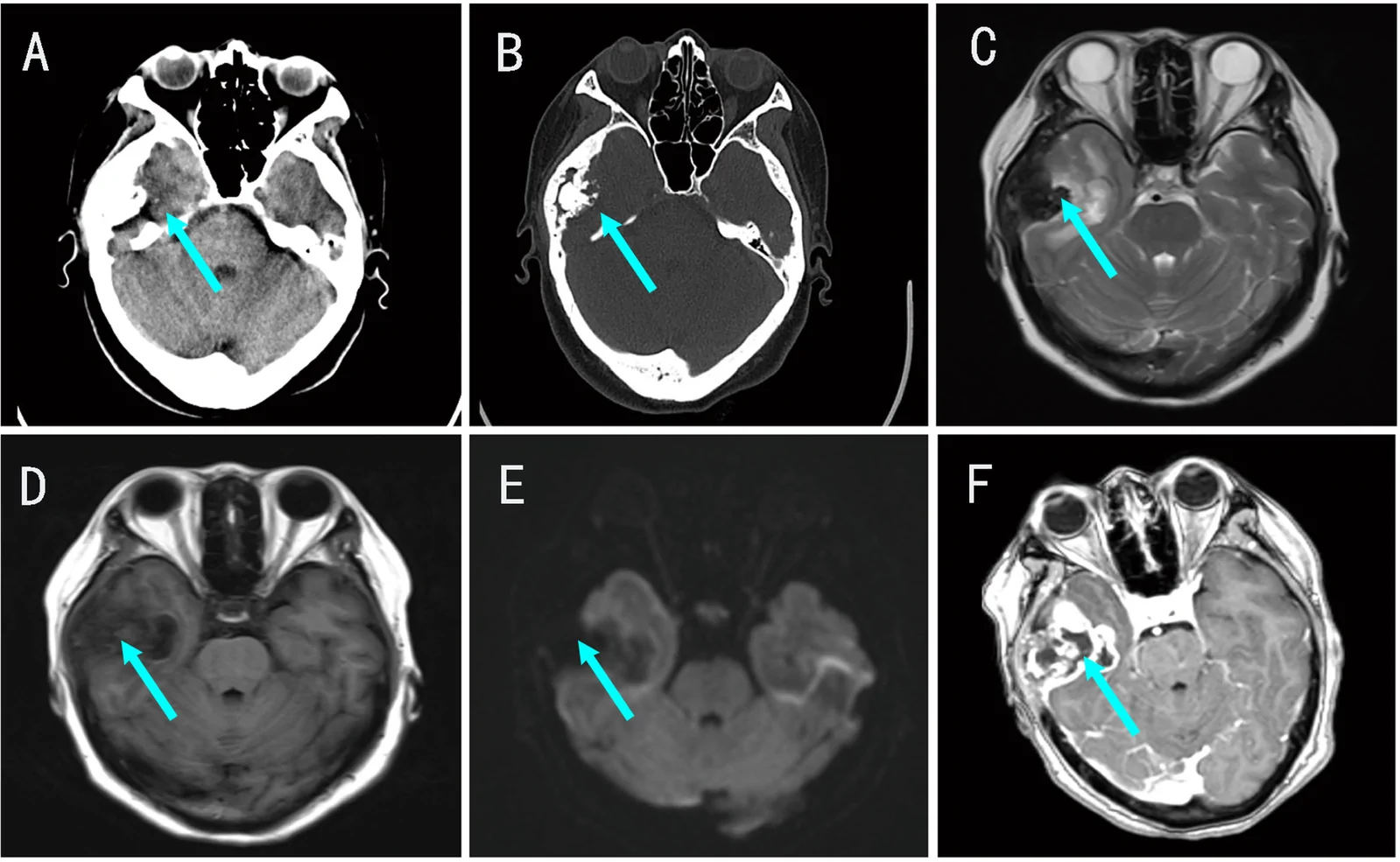
Preoperative CT and MRI findings. **(A)** Axial noncontrast CT (brain window) shows an irregular right temporal skull-base soft-tissue mass with intracranial extension and mass effect on the adjacent temporal lobe. **(B)** Axial CT (bone window) demonstrates expansile mixed lytic and sclerotic destruction of the right temporal bone and middle cranial fossa floor, with cortical disruption and residual bony septa. **(C)** Axial T2-weighted MRI shows heterogeneous hyperintensity and adjacent temporal-lobe edema. **(D)** Axial T1-weighted MRI shows predominantly iso- to mildly hypointense signal. **(E)** Axial diffusion-weighted imaging shows no convincing diffuse diffusion restriction. **(F)** Axial contrast-enhanced T1-weighted MRI demonstrates irregular peripheral and nodular enhancement surrounding central nonenhancing necrotic or cystic change. These findings document radiologic bone destruction but do not constitute histologic proof of osseous invasion.

### 2.2 Pathologic assessment

The main specimen measured 3.5 × 2.7 × 2.7 cm; additional tumor fragments measured up to 2.7 cm, and submitted bone fragments measured up to 3.7 cm. Histologic examination demonstrated a spindle-cell neoplasm with a patternless architecture and variably collagenous stroma. Areas of increased cellularity, cytologic atypia, scattered tumor giant cells, myxoid change, focal hemorrhage, and focal necrosis were present. Mitotic activity was documented as 4 mitoses per 10 high-power fields (HPF). Review of the available histologic sections by two pathologists showed no morphologically distinct high-grade sarcomatous component or convincing abrupt transition.

Immunohistochemistry showed nuclear STAT6 expression and weak, partial CD34 expression. The tumor was also positive for BCL-2, SMA, EMA, CD99, vimentin, MDM2, and weak progesterone receptor expression, whereas S100, desmin, AE1/AE3, GFAP, SOX10, myogenin, and SSTR2 were negative. The Ki-67 labeling index was approximately 40%. MDM2 immunoreactivity was interpreted cautiously as a nonspecific ancillary finding, because immunohistochemical expression alone does not establish MDM2 gene amplification (Figure 2).

**Figure 2:**
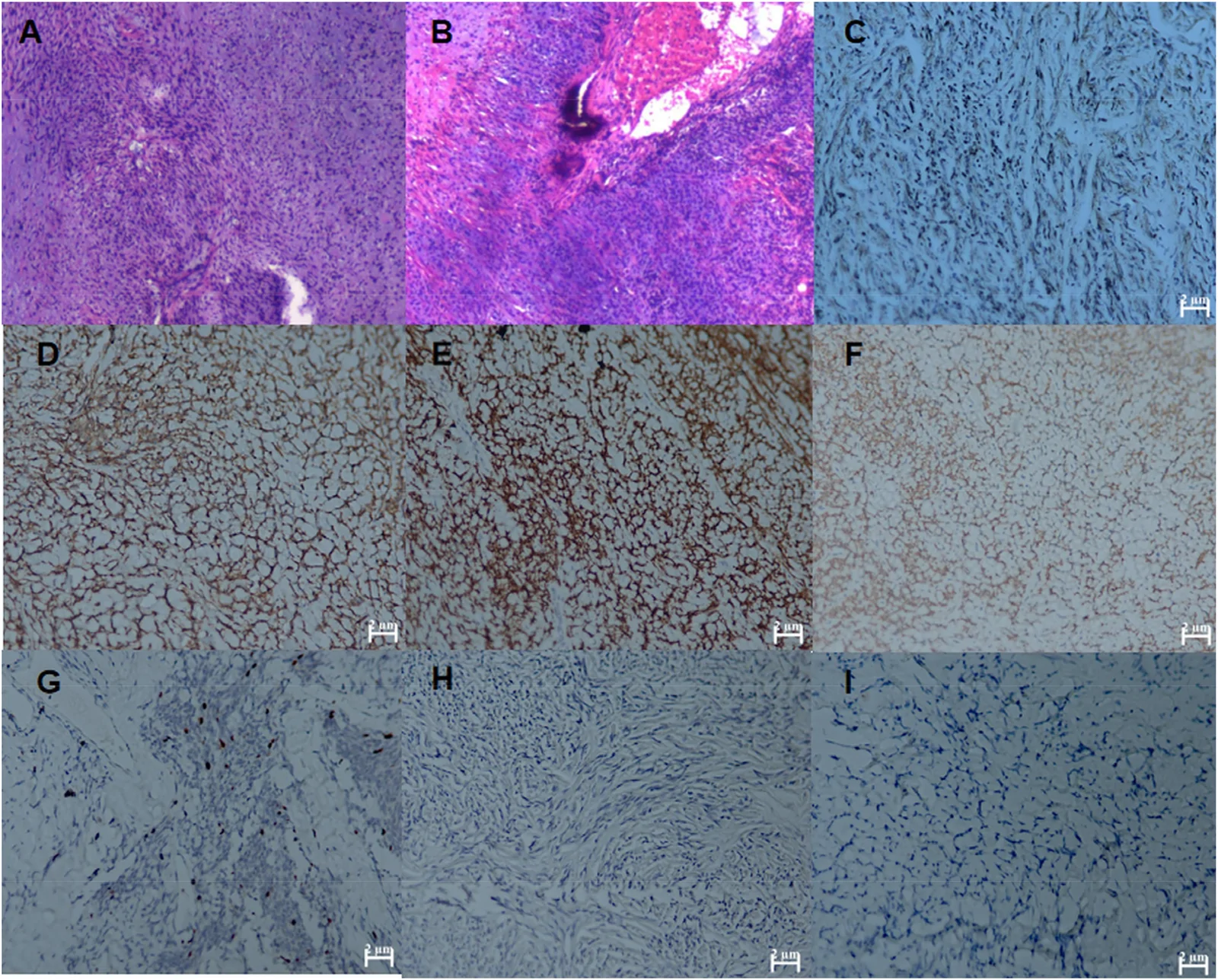
Histopathologic and immunohistochemical findings of the solitary fibrous tumor. **(A,B)** Hematoxylin and eosin-stained sections showing a spindle-cell neoplasm with a patternless architecture and areas of variable cellularity and cytologic atypia; review of the available sections showed no morphologically distinct high-grade sarcomatous component. **(C)** Nuclear STAT6 expression. **(D)** Weak and partial CD34 expression. **(E)** BCL-2 positivity. **(F)** CD99 positivity. **(G)** Increased Ki-67 labeling index (approximately 40%). **(H)** Negative S100 staining. **(I)** Negative GFAP staining. Representative images were acquired at 100× total magnification.

Applying the 2021 WHO Classification of Tumors of the Central Nervous System to the available pathologic data, the documented mitotic activity of 4 mitoses/10 HPF corresponds most closely to CNS WHO grade 1 (4). The microscope field diameter or field area used for the documented mitotic assessment was not recorded, and the count therefore could not be reliably converted to mitoses/mm^2^. Focal necrosis was present, although its proportion could not be quantified retrospectively. These limitations were considered when interpreting the grade.

Histologic examination of the submitted bone fragments showed no tumor involvement. Nevertheless, preoperative CT demonstrated marked destructive changes of the adjacent temporal bone, and intraoperative findings confirmed involvement of the abnormal temporal skull-base bone and tegmen tympani. Because a direct tumor–bone interface was not demonstrated histologically, the osseous findings are described as radiologic bone destruction with intraoperative osseous involvement**,** rather than histologically proven bone invasion.

### 2.3 Therapeutic intervention

The patient underwent right frontotemporal craniotomy and microsurgical resection under general anesthesia. Preoperative endovascular embolization was not performed. Although intracranial SFTs may be hypervascular, the anticipated intraoperative bleeding was considered surgically controllable based on the available preoperative imaging and the planned microsurgical approach; therefore, preoperative embolization was not pursued. No obvious major-vessel encasement or direct vascular involvement was identified preoperatively. Intraoperative neurophysiological monitoring was used during tumor removal. No major vessel was found to require reconstruction or sacrifice, and the estimated blood loss was less than 50 mL.

The squamous temporal bone was visibly abnormal and thickened. After elevation of the bone flap and drilling of the abnormal temporal bone, a dural-based tumor was identified along the middle cranial fossa floor. The dural attachment was divided and the extradural component was removed. The lesion showed intraoperative involvement of the temporal bone and tegmen tympani and extended toward the middle ear; the ossicular chain remained intact. The intradural component was then removed piecemeal under microscopic visualization. Because the lesion crossed multiple skull-base compartments and the arachnoid interface was disrupted, *en bloc* oncologic resection with histologically assessable negative margins was not feasible. Gross-total macroscopic removal was judged to have been achieved intraoperatively, but microscopic margin status remained unassessable.

The resulting middle cranial fossa floor/tegmen defect was reconstructed with autologous temporalis muscle. No postoperative cerebrospinal fluid leak or other major surgery-related complication was documented during the index hospitalization.

### 2.4 Follow-up and outcomes

An early postoperative MRI obtained during the index hospitalization showed removal of the preoperative enhancing mass, reduced mass effect, and expected T2-weighted signal change around the operative cavity. Linear and patchy enhancement was present along the surgical bed, adjacent dura, and soft tissues, without definite nodular or mass-like residual enhancement (Figure 3).

**Figure 3:**
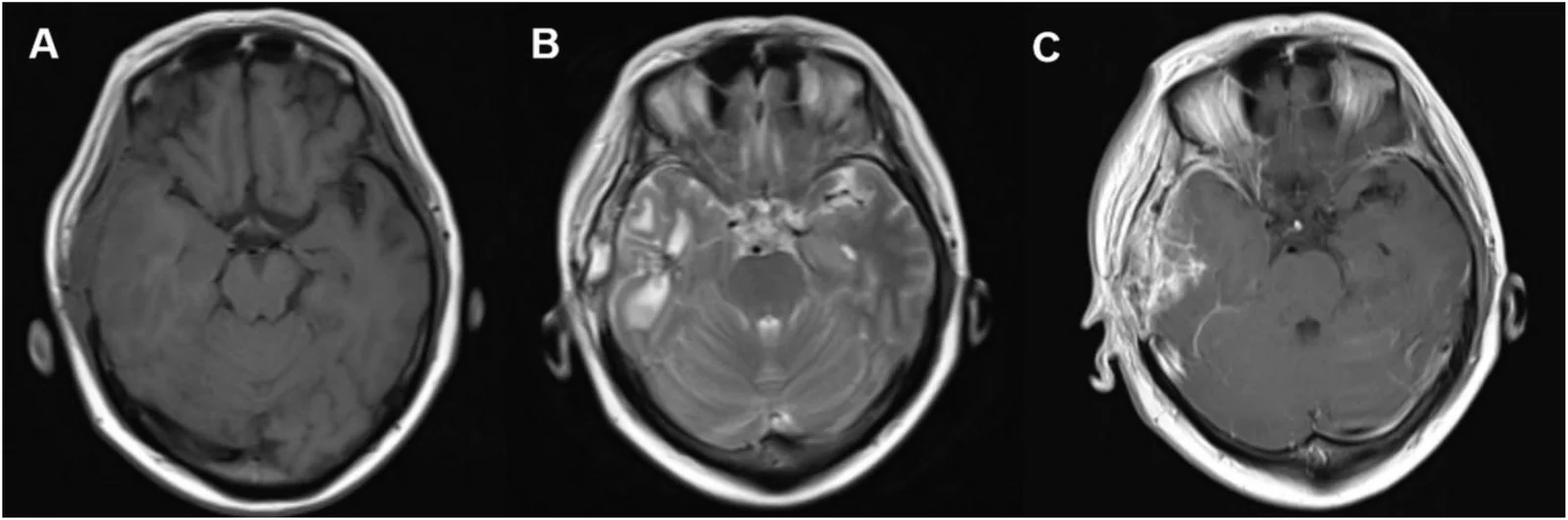
Early postoperative MRI findings. **(A)** Axial T1-weighted MRI demonstrates the right temporal skull-base operative cavity after tumor resection. **(B)** Axial T2-weighted MRI shows expected postoperative signal change and reduced mass effect on the adjacent temporal lobe. **(C)** Axial contrast-enhanced T1-weighted MRI shows no definite nodular or mass-like residual enhancement; thin linear and patchy enhancement along the operative bed is favored to represent postoperative change.

Because the lesion had been removed piecemeal and microscopic margins were unassessable, postoperative radiotherapy was discussed and the patient was advised to attend the radiation oncology clinic for further evaluation and adjuvant treatment. The patient declined postoperative radiotherapy and elected surveillance. No postoperative systemic antitumor therapy was administered.

Follow-up was completed at 31 months. At postoperative month 18, telephone follow-up indicated that MRI performed at an outside hospital reportedly showed tumor recurrence. At month 27, the patient reported further enlargement of the recurrent lesion on repeat outside-hospital imaging. The corresponding imaging studies were not available for review at our institution. During follow-up, the patient also reported that examinations performed at the outside hospital showed no evidence of metastatic disease at other sites, including the lungs, liver, or skeleton.

After recurrence was reported, further local treatment options, including repeat surgery and radiotherapy, were discussed. During later telephone follow-up, the patient's family expressed concern that she might not tolerate another major surgical intervention. No contemporaneous Karnofsky Performance Status or ECOG Performance Status was available. Given these concerns and the available follow-up information, continued clinical and imaging follow-up was recommended. No new focal neurologic deficit was reported during telephone follow-up, although the absence of an in-person examination limits objective assessment.

The clinical course and key diagnostic and therapeutic milestones are summarized in Table 1.

**Table 1:** Clinical timeline.

Relative time	Clinical course
Approximately 1 day before admission	Acute right facial weakness developed.
Admission	Neurological examination involved both upper and lower right facial muscles and was considered consistent with peripheral facial nerve palsy; no additional cranial-nerve deficit was documented, and a formal House-Brackmann grade was not recorded. CT demonstrated mixed destructive change of the right temporal bone and middle cranial fossa floor. MRI showed a 46 × 38 × 36 mm lobulated mass with heterogeneous signal, irregular peripheral/nodular enhancement, central necrotic or cystic change, and adjacent temporal-lobe edema. Initial systemic imaging showed no distant metastasis.
Day 5	Right frontotemporal craniotomy and microsurgical resection were performed with intraoperative neurophysiological monitoring. Preoperative embolization was not performed; estimated blood loss was <50 mL. The dural-based lesion had extradural and intradural components, involved the tegmen tympani, and extended toward the middle-ear region. Gross-total macroscopic removal was achieved, but microscopic margins were unassessable. The skull-base defect was reconstructed with autologous temporalis muscle.
Postoperative pathology	Histology and immunohistochemistry supported SFT: 4 mitoses/10 HPF, focal necrosis, nuclear STAT6 positivity, weak partial CD34 expression, MDM2 immunoreactivity, and Ki-67 ∼40%. Review of the available sections showed no morphologically distinct high-grade sarcomatous component. Under the 2021 CNS WHO framework, the documented mitotic count corresponds most closely to grade 1.
Early postoperative period	MRI during the index hospitalization showed no definite nodular or mass-like residual enhancement. No postoperative CSF leak or major surgical complication was documented. Adjuvant radiotherapy was recommended, but the patient declined and chose surveillance.
Postoperative month 18	Telephone follow-up indicated that outside-hospital MRI reportedly showed tumor recurrence.
Postoperative month 27	Telephone follow-up indicated further enlargement on repeat outside imaging. The patient reported outside examinations of the lungs, liver, and skeleton without detected metastasis.
Postoperative month 31	Final follow-up was completed by telephone. During later follow-up, the family expressed concern that the patient might not tolerate another major surgical intervention; continued clinical and imaging surveillance was recommended. No contemporaneous KPS/ECOG assessment was available.

## 3 Discussion

The principal value of this case is not simply the rarity of a temporal skull-base SFT, but the discordance between destructive skull-base anatomy, a documented mitotic count within the CNS WHO grade 1 range, and relatively early reported postoperative recurrence. The tumor crossed the dural boundary, involved the tegmen tympani, extended toward the middle-ear region, and produced marked temporal-bone destruction, while the recorded mitotic count remained below the threshold for CNS WHO grade 2. This combination highlights three practical issues relevant to skull-base SFT: how radiologic and operative behavior should be interpreted when formal histologic grade is low, how gross-total macroscopic resection should be understood when a tumor is removed piecemeal across complex compartments, and how postoperative surveillance should be planned when grade alone may not fully capture clinical behavior.

Temporal skull-base SFTs frequently overlap radiologically with meningioma, schwannoma, and other hypervascular spindle-cell tumors, and imaging is often more useful for defining surgical anatomy than for establishing tumor lineage (2, 3). In a recently reported giant communicating temporal skull-base SFT, the lesion extended through the foramen ovale into the infratemporal fossa and was managed with a combined craniofacial approach, with no recurrence reported at 22 months (2). The present case followed a different anatomic pattern: the lesion was centered on the middle cranial fossa floor, crossed intradural and extradural compartments, involved the tegmen tympani, extended toward the middle-ear region, and was associated with destructive temporal-bone change. This distinction is clinically important because the pattern of extension influences surgical exposure, reconstruction, and the feasibility of obtaining assessable margins. Conversely, destructive CT findings should not automatically be translated into histologically proven bone invasion. In this case, submitted bone fragments were negative despite marked radiologic destruction and abnormal bone observed intraoperatively, illustrating the limitations of sampling a complex skull-base tumor-bone interface. The most defensible description is therefore radiologic bone destruction with intraoperative osseous involvement, without histologic proof of bone invasion.

Pathologic interpretation also requires separation of diagnosis from risk stratification. Nuclear STAT6 expression, together with the characteristic spindle-cell morphology and patternless collagenous architecture, strongly supports SFT and is a reliable surrogate for NAB2::STAT6 fusion in the appropriate setting (5–8). CD34 is supportive but not specific. MDM2 immunoreactivity in this tumor should be regarded as an ancillary, nonspecific finding rather than as evidence of an alternative MDM2-amplified neoplasm in the absence of molecular confirmation. Applying the 2021 CNS WHO framework to the available data, the recorded 4 mitoses/10 HPF corresponds most closely to grade 1 (4). Focal necrosis and a Ki-67 labeling index of approximately 40% do not by themselves alter the grade assignment based on the documented mitotic criteria, but they remain biologically relevant observations. Recent clinical data indicate that WHO grade and proliferative activity are associated with outcome while also showing that recurrence is not confined to high-grade tumors (9). Accordingly, a grade 1 designation should be understood as a histopathologic category rather than a guarantee of indolent behavior. In this case, the absence of a documented hotspot designation and microscope field area further limits how precisely the recorded mitotic count can be compared with contemporary quantitative practice.

The surgical course illustrates a second source of risk that is not captured by grade. At the skull base, gross-total resection is often a macroscopic operative judgment rather than an R0 oncologic resection. Because this lesion crossed the dura and skull base and was removed piecemeal, microscopic margins could not be assessed even though early postoperative MRI showed no definite nodular or mass-like residual enhancement. This distinction is important when postoperative radiotherapy is considered. Retrospective studies have reported improved local control or progression-free outcomes with postoperative radiotherapy in selected intracranial or meningeal SFTs, particularly after subtotal resection, recurrence, or other adverse features, although the benefit after gross-total resection of a grade 1 tumor remains uncertain and the available evidence is heterogeneous (10–13). In this patient, postoperative radiotherapy was recommended because of the skull-base location, piecemeal removal, and unassessable microscopic margins. The subsequent recurrence is clinically important, but a single case cannot establish that omission of radiotherapy caused recurrence or that adjuvant radiotherapy would have prevented it.

The reported recurrence at 18 months, with further enlargement by 27 months, was relatively early compared with the delayed recurrences often emphasized in SFT literature and therefore reinforces the need for both early and prolonged surveillance. At the same time, these follow-up findings were obtained at an outside hospital and the original images were unavailable, so the recurrent volume, exact location, growth kinetics, and suitability for stereotactic radiosurgery could not be independently assessed. Stereotactic radiosurgery can provide local control for selected residual or recurrent intracranial SFTs, but candidacy depends on lesion size, location, prior treatment, and proximity to critical structures (14). More broadly, long-term surveillance is justified because intracranial SFT may recur locally or metastasize extracranially after prolonged intervals (9, 15). In the present case, the patient reported that outside examinations of the lungs, liver, and skeleton showed no metastatic disease. Thus, the follow-up lesson is not that every grade 1 tumor requires the same intervention, but that surveillance intensity and salvage planning should be individualized according to anatomy, margin certainty, pathologic features, and patient-specific treatment considerations.

The strength of this report is the integrated correlation of preoperative CT and MRI, operative anatomy, histomorphology, immunophenotype, early postoperative imaging, and 31 months of longitudinal follow-up. Several limitations should be considered. Molecular confirmation of NAB2::STAT6 fusion and MDM2 amplification was not available; the documented mitotic assessment lacked a recorded field area and explicit hotspot designation; the extent of necrosis could not be quantified retrospectively; piecemeal resection precluded microscopic margin assessment; formal otologic and audiometric testing was not performed; and the outside studies documenting recurrence and later enlargement were unavailable for central review. These limitations reduce the precision of grading and longitudinal assessment and should be considered when interpreting the observed clinicopathologic discordance.

Taken together, this case suggests a practical framework in which diagnosis, grading, resectability, and recurrence risk are considered as related but distinct questions. STAT6-supported SFT diagnosis and CNS WHO grading provide the pathologic foundation, whereas skull-base extension, margin assessability, and postoperative imaging define the practical local-control problem. For lesions with destructive growth or unassessable margins, postoperative radiotherapy may warrant individualized multidisciplinary discussion, and long-term surveillance remains important even when the documented mitotic count falls within the grade 1 range. Additional molecularly annotated series are needed to determine which histologic and clinical features best identify the subset of low-grade CNS SFTs at greatest risk of recurrence.

## 4 Patient perspective

The patient reported improvement in her right facial weakness after surgery. Postoperative radiotherapy was discussed and recommended, but she did not undergo adjuvant treatment and continued surveillance. After recurrence was reported during outside-hospital follow-up, further local treatment options were discussed. During later telephone follow-up, her family expressed concern that she might not tolerate another major surgical intervention, and continued clinical and imaging follow-up was recommended.

## Data Availability

The original contributions presented in the study are included in the article/Supplementary Material, further inquiries can be directed to the corresponding authors.
